# Effects of *Cudrania tricuspidata* on anti‐senescence in high glucose‐treated endothelial cells via the Akt/p53/p21 pathway

**DOI:** 10.1002/fsn3.1885

**Published:** 2020-09-15

**Authors:** Gi Dae Kim, Seonghee Park

**Affiliations:** ^1^ Department of Food and Nutrition Kyungnam University Changwon‐si Republic of Korea; ^2^ Department of Biological Science Sookmyung Women's University Seoul Republic of Korea

**Keywords:** Akt/p53/p21, *Cudrania tricuspidata*, endothelial cell, senescence

## Abstract

The roles of *Cudrania tricuspidata* (CT) in the prevention of senescence and the underlying mechanisms have not been elucidated. In a high glucose (HG)‐induced senescent endothelial cell (EC) culture, CT (20 µg/ml) reduced the number of senescence‐associated β‐galactosidase‐positive cells by 8.3% compared with the control group and increased the expression of p‐Sirt1 by more than twofold compared with the control group. Moreover, 20 μg/ml CT treatment doubled the activity of p‐Akt, which was inhibited by HG, compared with the control group. In addition, CT treatment decreased the expression of p53, p21, and Rb, which was increased by HG. Overall, CT delays HG‐induced senescence via the Akt/p53/p21 pathway, suggesting its potential as a functional agent for the protection of ECs.

## Graphical abstract

*Cudrania tricuspidata* delays high glucose‐induced senescence through the Akt/p53/p21 pathway. *Cudrania tricuspidata* may be a functional agent for the protection of endothelial cells.

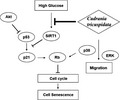

## 1 INTRODUCTION

Vascular complications significantly contribute to the incidence of diabetes mellitus and the associated mortality (Brownlee, 2005). The major causes of vascular complications in diabetes are endothelial dysfunction and hyperglycemia related to damaged neovascularization (Sheetz & King, 2002). Endothelial dysfunction is a major cause of atherosclerosis development; therefore, it is important that the vessels are healthy to lower the risk of cardiovascular complications associated with diabetes mellitus (Kinlay, Libby, & Ganz, 2001). Endothelial cell (EC) senescence is involved in vascular aging (Yin & Pickering, 2016), and senescence is an independent risk factor for vascular diseases. Thus, EC senescence promotes vascular dysfunction (Bachschmid et al., 2013).

Cellular senescence is regulated by various molecular signaling pathways. The known biomarkers of cellular senescence include the p53 (tumor suppressor), p21 (cell cycle kinase‐dependent inhibitor), and retinoblastoma (Rb; cell cycle regulator) proteins. Moreover, the p53/p21/Rb pathway is activated in response to senescence (Muller, 2009). The tumor suppressor gene, *p53*, is involved in several aspects of cell biology, including cell proliferation, senescence, and death. p53 responds to signals by initiating the first step of irreversible cell cycle arrest. Recently, the Akt/p53/p21/Rb pathway was shown to play an important role in the regulation of cellular senescence (Feng et al., 2016). Additionally, senescence‐associated beta‐galactosidase (SA‐β‐gal) is a representative biomarker of senescence (Sikora, Bielak‐Zmijewska, & Mosieniak, 2014).

*Cudrania tricuspidata* (CT) is used in Korean traditional medicine to treat inflammation, gastritis, tumors, and hepatocellular damage (Chang et al., 2008). The use of its leaves, fruits, and roots is described in the study of traditional medicine in Donguibogam, a classic Eastern medicine book used in various countries, including Korea, China, and Japan. The roots and leaves of this perennial herb contain bioactive substances that exhibit anti‐cancer, anti‐oxidant, and hypoglycemic effects. The root bark of CT is reported to have anti‐platelet (Park et al., 2006), anti‐inflammatory (Jeong, Lee, & Kim, 2009), anti‐oxidant (Lee, Kim, Lee, Ham, & Whang, 2009), neurotherapeutic (Kwon et al., 2016), hepatocellular protective (Tian, Kim, Cui, & Kim, 2005), and cytotoxic (Lee et al., 2005) effects. Although several studies have investigated CT, there is little research on its ability to prevent EC senescence. Therefore, in this study, we established a high glucose (HG)‐induced senescent model of human umbilical vein endothelial cells (HUVECs) and examined the molecular mechanism to determine the anti‐senescence effects of CT.

## 2 MATERIALS AND METHODS

### 2.1 Materials and reagents

CT was obtained from Dr. Park (Kyungnam University, Changwon‐si, Republic of Korea), and CT extraction and separation were performed as previously reported (Shon et al., 2014). An SA‐β‐gal kit was purchased from Abcam Inc. (Cambridge, MA, USA). P‐p38, p53, p‐21, p‐silent information regulator 1 (Sirt1), Sirt1, and p‐Akt antibodies were purchased from Cell Signaling Technology (Danvers, MA, USA). Β‐Actin, p‐extracellular signal‐regulated kinase (ERK), and Rb antibodies were obtained from Santa Cruz Biotechnology (Dallas, TX, USA). Horseradish peroxidase (HRP)‐conjugated anti‐mouse and anti‐rabbit antibodies were procured from GeneTex Inc. (Irvine, CA, USA).

### 2.2 Endothelial cell culture

HUVECs were obtained from the American Type Culture Collection (Manassas, VA, USA) and cultured in endothelial growth medium (EGM‐2; Lonza, Walkersville, MD, USA) supplemented with 10% fetal bovine serum (FBS) under 5% CO_2_ at 37°C (Kim et al., 2008). ECs were cultured in EGM‐2 or low glucose (LG; 6 mmol/L) or HG (30 mmol/L) medium with or without CT at different concentrations (5, 10, and 20 μg/ml) for 48 hr.

### 2.3 Cell viability assay

Cells were seeded into a 96‐well plate (5 × 10^3^ cells/well). When the cells reached 70% confluence, they were cultured in fresh 2% FBS containing various concentrations of CT at 37°C for 24–72 hr, and then treated with MTT (5 mg/ml) solution for 4 hr. The resulting formazan deposits in each well were dissolved in dimethyl sulfoxide, and the absorbance of the sample was measured at 570 nm using a microplate reader (Molecular Devices, Sunnyvale, CA, USA).

### 2.4 Migration assay

The experimental process followed a previously method (Oh, Kim, Kim, & Lee, 2017). Briefly, cells were cultured until they reached 70% confluence in 6‐well plates precoated with 0.1% gelatin. Cell monolayers were wounded by scratching with a 0.2‐ml pipette tip, followed by the addition of fresh medium containing various concentrations of CT. The cells were maintained for 24–48 hr. Images were captured using a light microscope (Olympus Optical Co., Ltd., Tokyo, Japan) at the point of complete migration. The migrated cells were manually counted using an advanced program.

### 2.5 Transwell invasion assay

The motility of ECs was determined using transwell plates (Corning Inc., Corning, NY, USA) with a pore size of 8 μm (Yi et al., 2008). The inserts of the transwell plates were coated with 0.2% gelatin for 30 min and washed three times with phosphate‐buffered saline (PBS). Then, fresh endothelial basal medium with 20 ng/ml vascular endothelial growth factor (VEGF) was added into the lower chamber and ECs were seeded in the top chamber. The cells were then treated with CT for 8–12 hr. The chamber membrane containing the migrated cells was fixed with 4% paraformaldehyde and stained with hematoxylin in the dark for 10 min. Images were captured using a microscope, and the migrated cells were manually counted.

### 2.6 SA‐β‐gal staining

The senescence analysis was performed using the SA‐β‐gal kit in accordance with the manufacturer's instructions. The ECs were fixed in β‐gal fixative for 5 min, washed with PBS, and stained using β‐gal solution at 37°C. SA‐β‐gal‐positive cells were examined and quantified under a microscope.

### 2.7 Cell cycle analysis

Cells were fixed with 70% ethanol overnight at 4°C, washed, and stained with 50 μg/ml propidium iodide (PI) and 50 μg/ml RNase A for 1 hr in the dark. Cells were analyzed using the FACSCalibur flow cytometer (Becton Dickinson, San Jose, CA, USA). The results were analyzed for cell cycle distribution using Cell Quest software (Becton Dickinson).

### 2.8 Protein extraction and Western blotting

The cells were treated with protein extracts containing protease inhibitors and phosphatase inhibitors for 10 min at 4°C. The total protein in culture supernatants was quantified using the Bradford assay. Cell lysates were resolved using sodium dodecyl sulfate‐polyacrylamide gel electrophoresis. The samples were transferred onto polyvinylidene fluoride membranes (Millipore, Bedford, MA, USA) at 100 V for 60–100 min. The membranes were incubated with primary antibodies followed by secondary antibodies (HRP‐conjugated goat anti‐rabbit or anti‐mouse antibodies). Bands were detected using enhanced chemiluminescence detection reagents (Intron Biotechnology Inc., Seongnam, South Korea) and quantified using ImageJ software (National Institutes of Health, Bethesda, MD, USA).

### 2.9 Statistical analysis

All data are expressed as average ± *SD*, and statistical significance was determined using appropriate post hoc test and deviation analysis. Calculations were performed using SPSS for Windows (v.23.0; IBM Corp., Armonk, NY, USA), and the results with *p‐*values < .05, .01, and .001 were considered statistically significant.

## 3 RESULTS

### 3.1 Effect of CT on the viability of ECs

**FIGURE 1:**
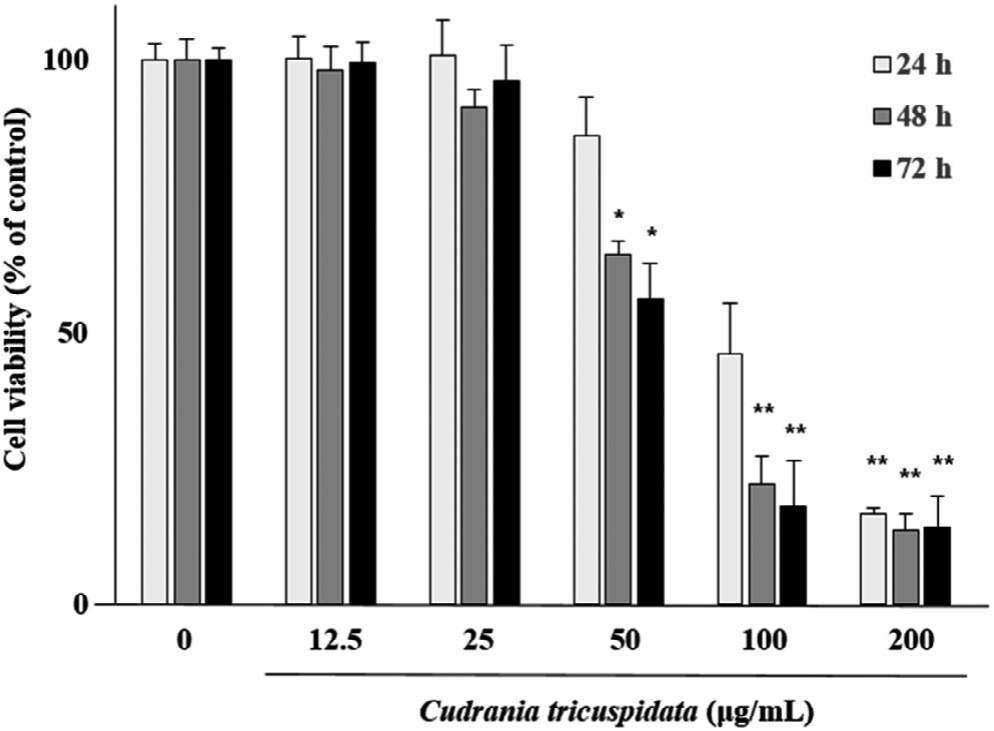
Effects of *Cudrania tricuspidata* (CT) on endothelial cell (EC) viability. ECs were treated with CT (0–200 μg/ml) for 72 hr. Cell viability is expressed as the percentage of cultured viable cells in medium without CT. Bars represent mean ± *SD*. *p < .05 and **p < .01

The viability of ECs treated with CT at nontoxic concentrations (0, 12.5, 25, 50, 100, and 200 µg/ml) for 72 hr was evaluated. The viability of ECs was significantly reduced after 48 hr of treatment with CT at concentrations of > 50 µg/ml (*p* < .05, *p* < .01) (Figure 1). Therefore, the subsequent experiments were performed with CT at nontoxic concentrations (i.e., ≤25 μg/ml).

### 3.2 Characteristics of ECs treated with CT

**FIGURE 2:**
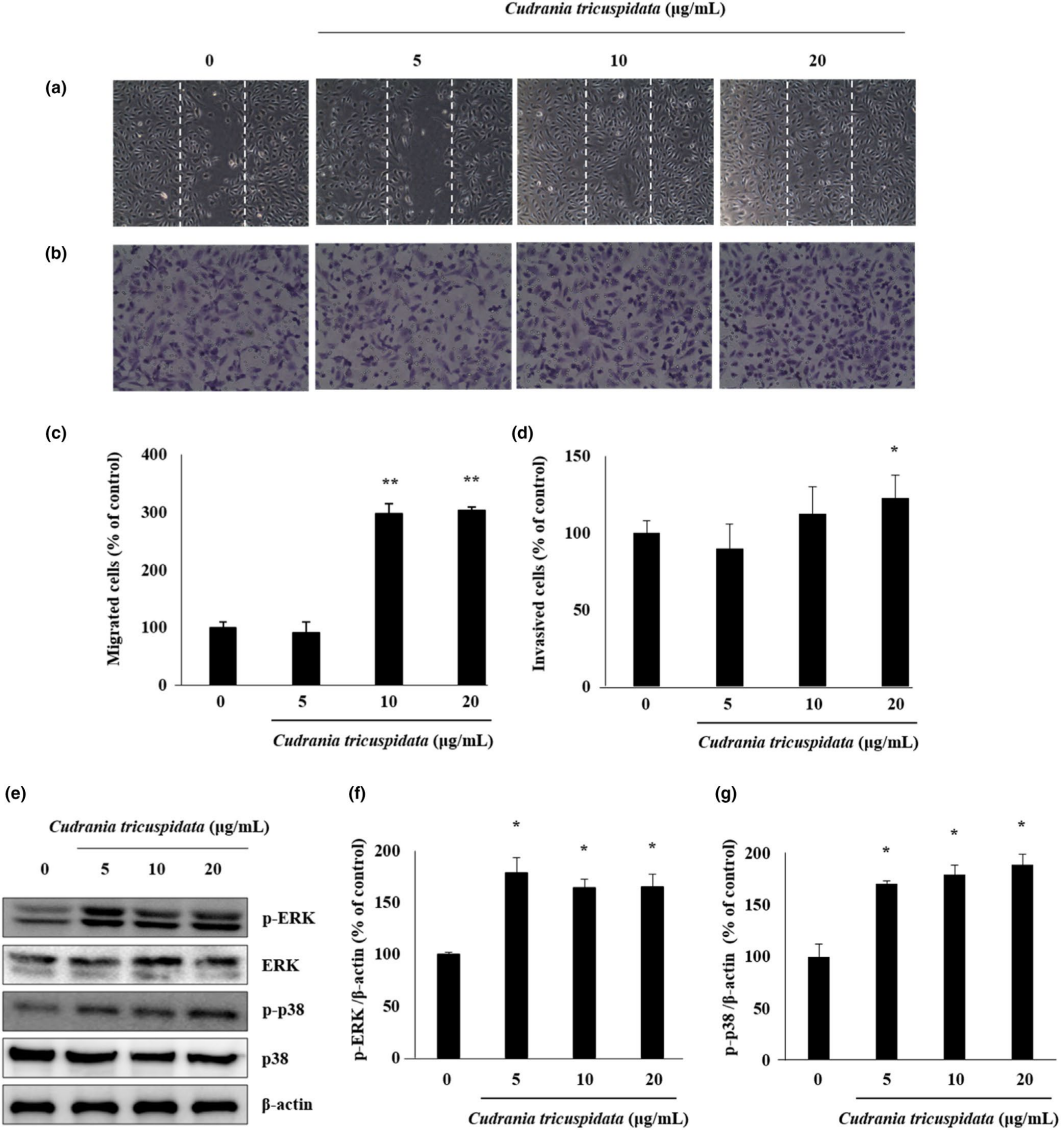
Effects of CT on the migration and invasion of ECs. (a and b) The images show cell migration and invasion in the control and CT treatment groups. (c and d) Cell migration and invasion are expressed as observed proportions in the control cells. (e) Representative blots of p‐ERK, ERK, p‐p38, and p38. (F, G) Quantitative analysis of both p‐ERK/β‐actin and p‐p38/β‐actin. Bars represent mean ± *SD*. *p < .05; **p < .01 versus the control group

The migration of ECs in response to VEGF is important during wound healing and invasion. The effect of CT on the migration of ECs was assessed using the wound‐healing assay. CT treatment (10 and 20 μg/ml) increased the migration of ECs approximately threefold compared with the control group (*p* < .01) (Figure 2a and c). Moreover, the invasion of VEGF‐induced ECs, one of the characteristics of these cells, significantly increased in the CT group (20 µg/ml) (*p* < .05) (Figure 2b and d). In the Western blot analysis, treatment with various concentrations of CT (5, 10, and 20 μg/ml) resulted in no difference in protein expression but significantly increased the level of p‐ERK and p‐p38 in ECs (*p* < .05) (Figure 2e–G).

### 3.3 Characteristics of senescence of ECs following glucose treatment

**FIGURE 3:**
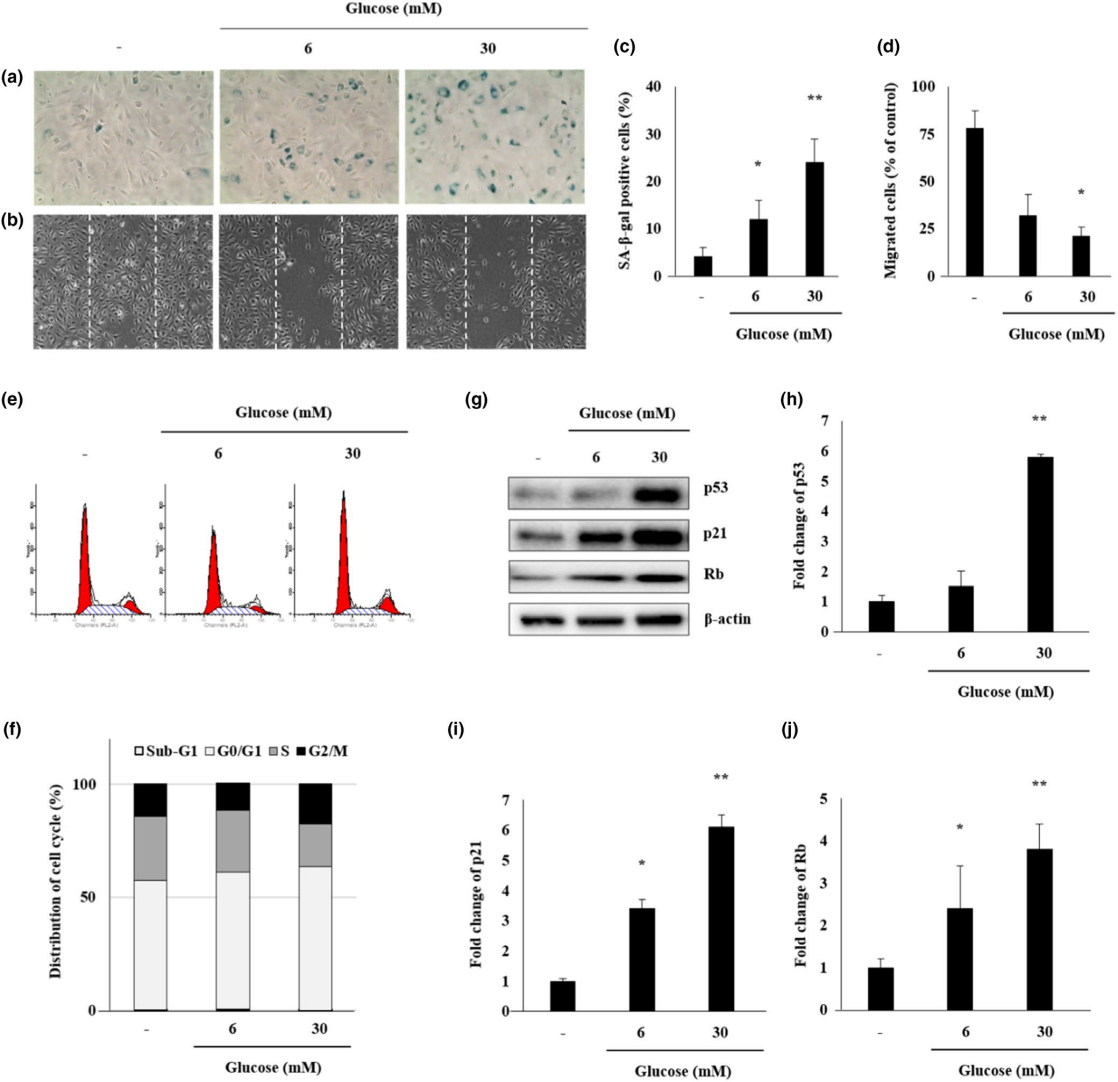
Characteristics of senescence induction in ECs with glucose treatment. (a and b) The images of senescence‐associated β‐galactosidase (SA‐β‐gal) staining and migration of ECs treated with medium alone, medium containing low glucose (LG; 6 mmol/L), or high glucose (HG; 30 mmol/L) for 48 hr. (c and d) The percentage of SA‐β‐gal‐positive cells and migrated cells in the different groups. (e) Cell cycle distribution was measured by flow cytometry using PI. (f) The percentage of cells in different cell cycle phases after ECs were treated with medium alone or medium containing LG or HG for 48 hr. (g) The protein levels of p53, p21, and Rb were determined by western blot analysis. (h–j) Quantification of p53, p21, and Rb relative to β‐actin. Bars represent mean ± *SD*. *p < .05; **p < .01 versus the control group

ECs were treated with glucose (6 and 30 mmol/L) for 48 hr to induce senescence. The number of SA‐β‐gal‐positive cells, a biomarker of senescent ECs, significantly increased following treatment with glucose (LG: 12.4%, HG: 24.2%; *p* < .05, *p* < .01, respectively) (Figure 3a and c). Moreover, in the senescence‐induced EC model, the treatment with HG significantly (HG: 21.7%, *p* < .05) reduced the migration of ECs (Figure 3b and d). The cell cycle is halted in senescent cells; therefore, we analyzed the cell cycle stages in senescent ECs treated with different glucose concentrations. G1 and G2 arrest occurred in cells treated with 30 mmol/L glucose for 48 hr (Figure 3e and f). We continued treatment with glucose to induce senescence in ECs and verified cell cycle arrest in the G1 and G2 phases. Based on these results, the proteins involved in cell cycle regulation were shown (Figure 3g). Glucose treatment, particularly at 30 mmol/L, significantly increased the concentration of proteins that regulate the cell cycle, such as p53, p21, and Rb (*p* < .01) by 5.8‐, 6.1‐, and 3.8‐fold, respectively, compared with the control group (Figure 3h–J).

### 3.4 Suppression of senescence in HG‐induced ECs by CT

**FIGURE 4:**
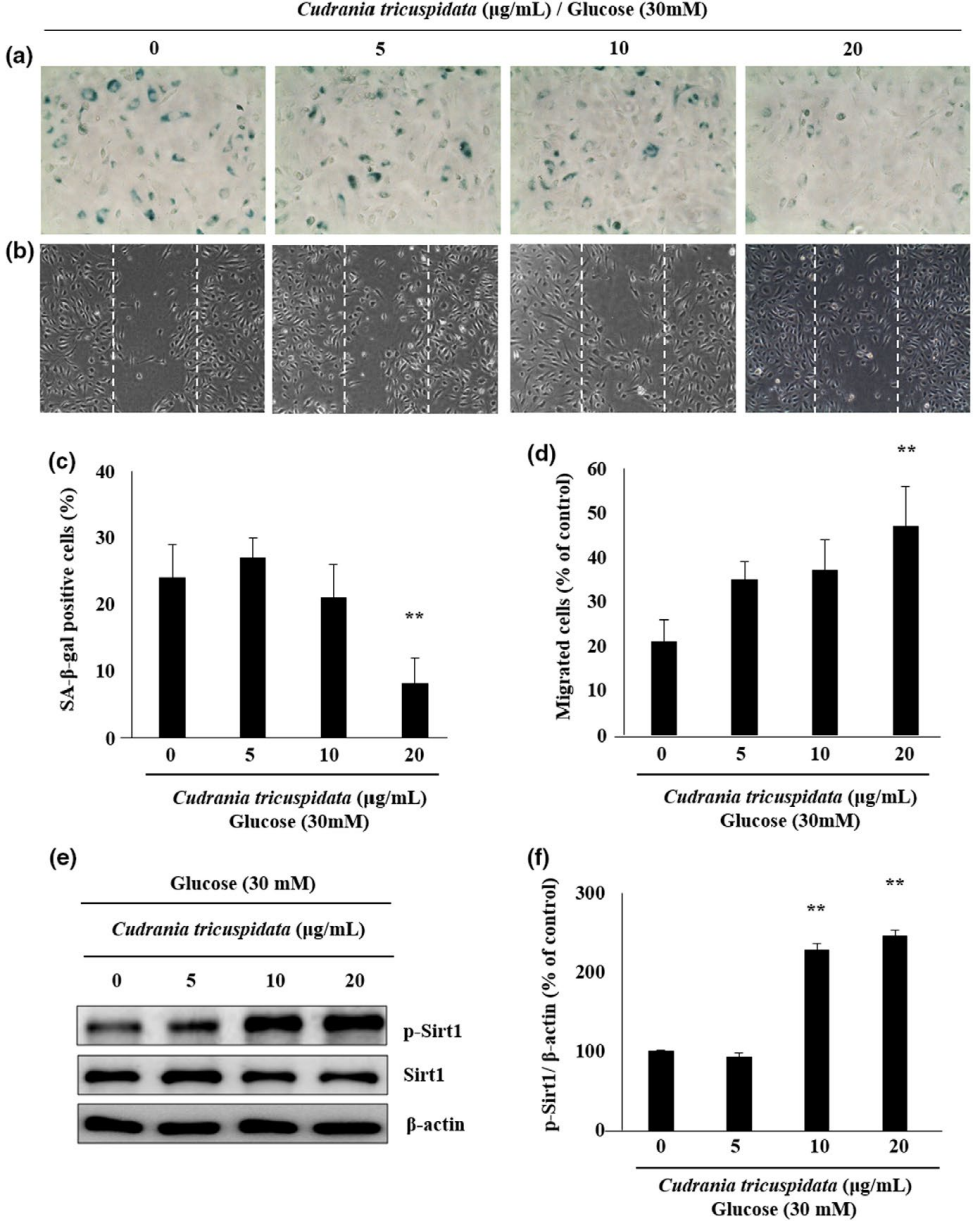
Effects of CT on the senescence of ECs cultured in the HG medium. (a and b) Representative images of SA‐β‐gal staining and migration of ECs treated with medium containing HG or medium containing HG and CT (5, 10, and 20 μg/ml) for 48 hr. (c and d) SA‐β‐gal‐positive cells and migrated cells in the treatment groups were calculated as a percentage of those in the control group. (E) Representative blots of p‐Sirt1 and Sirt1. (f) Quantification of p‐Sirt1 relative to Sirt1. The results are presented as mean ± *SD*. *p < .05; **p < .01 versus the control group

**FIGURE 5:**
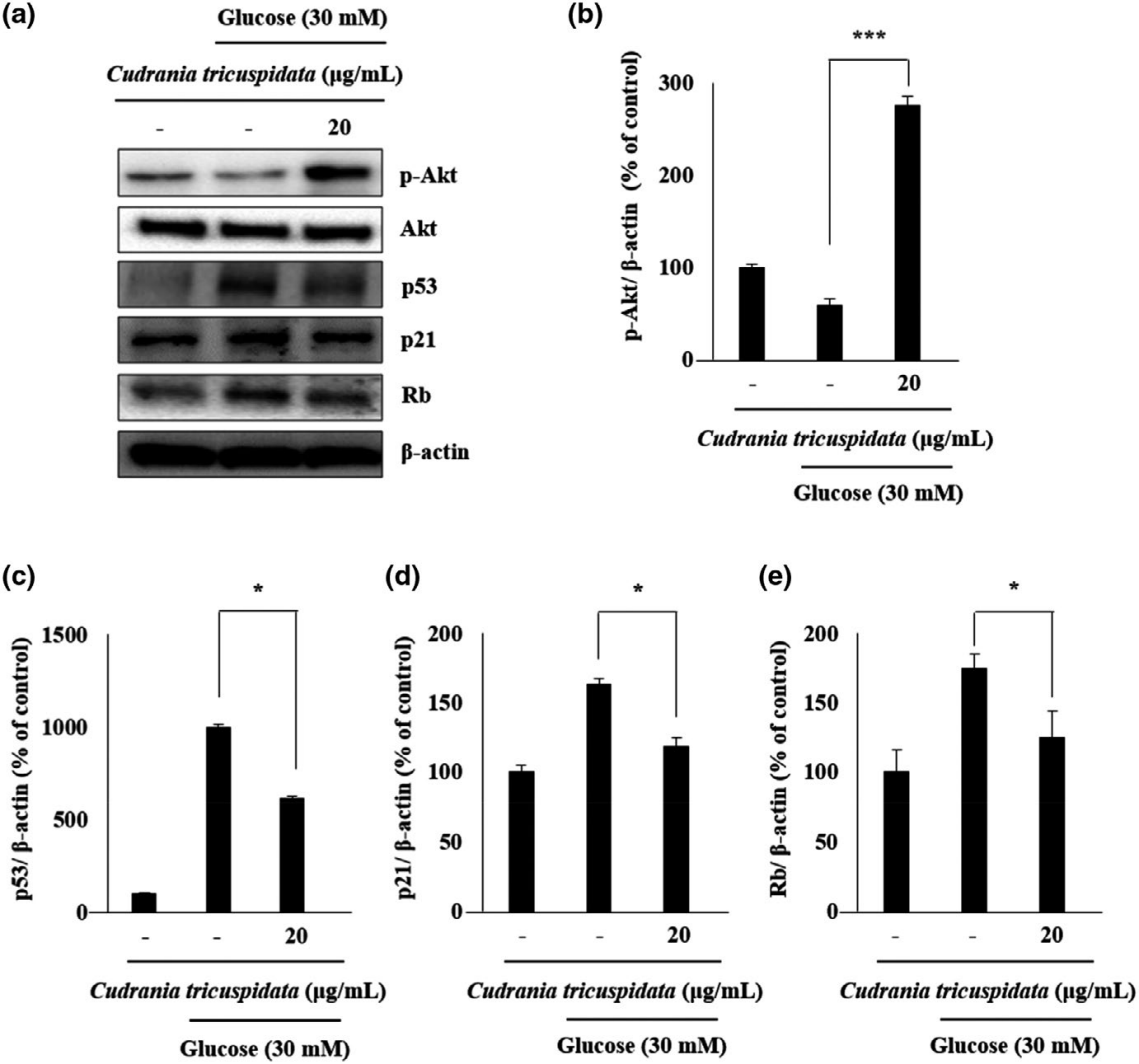
Effects of CT on the regulation of the Akt/p53/p21 signaling pathway in ECs exposed to HG. Cells were cultured in media alone, media containing HG, or media containing HG and 20 μg/ml CT for 48 hr, and then subjected to western blotting analysis. (a) Western blot analysis of p‐Akt, p53, p21, and Rb expression. (b–e) Quantification of p‐Akt, p53, p21, and Rb relative to β‐actin. Bars represent mean ± *SD*. *p < .05; ***p < .001 compared to HG group

To verify the suppression of senescence, the HG‐treated ECs were treated with different concentrations of CT. The results indicated a reduced tendency of senescence following treatment with 20 µg/ml CT. There was a significant reduction in the number of SA‐β‐gal‐positive cells (*p* < .01) by 8.3% compared with 24.5% in the control group (Figure 4a and c). EC migration increased following CT treatment. The migration of cells increased by 47.6% following CT treatment at 20 µg/ml, and this was significantly higher than that (21.3%) in the control group (*p* < .01) (Figure 4b and d). The suppression of SA‐β‐gal‐positive cells by CT treatment was confirmed in ECs. The expression of p‐Sirt1, a regulator of senescence, was analyzed to determine the effect of CT treatment in controlling Sirt1 expression. CT treatment (10 and 20 µg/ml) significantly increased the expression of p‐Sirt1 by more than twofold compared with the control group (*p* < .01) (Figure 4e and f). The effects of CT treatment on the expression patterns of proteins involved in the regulation of senescence were shown. Although senescence resulted in the suppression of p‐Akt activation, CT treatment (20 µg/ml) significantly increased the suppressed p‐Akt activity by threefold compared with HG group (*p* < .001) (Figure 5a and b). In addition, the expression of p53, p21, and Rb increased during senescence and significantly decreased (*p* < .05) following CT treatment at 20 µg/ml compared with HG group (Figure 5a, c–e).

## 4 DISCUSSION

CT leaves are rich in phenolic compounds, such as quercetin, kaempferol, myricetin, gallic acid, and flavonoids that exert various physiological effects (Jeong et al., 2010). In this study, we demonstrated that CT inhibits the increased expression of p53 (Orimo et al., 2009) and p21, which are markers of senescence induced by HG. In addition, SA‐β‐gal‐positive cells were inhibited and vascular EC migration was restored.

Sirt1 functions as an NAD^+^‐dependent histone deacetylase and a transcription factor for the expression of a variety of genes. As a multifunctional protein, Sirt1 has been shown to regulate cell proliferation, apoptosis, DNA damage repair, senescence, metabolism (Guarani & Potente, 2010), and longevity in response to caloric restriction in several organisms (Longo & Kennedy, 2006). Sirt1 is also an important transcription factor in intracellular signaling pathways (Xiong et al., 2013; Zhou et al., 2011). Sirt1 has been described as a major regulator of vascular EC homeostasis (Fry et al., 2015; Potente et al., 2007; Stein & Matter, 2011). Our results showed that, under HG conditions, the expression of Sirt1 and the proliferative and migratory capabilities of ECs decreased. The senescence of ECs plays a critical role in the pathogenesis of cardiovascular diseases and is mainly caused by aging, diabetes, and stress (Burton, 2009). Several senescence markers are available to identify senescent cells, among which the most widely used is SA‐β‐gal activity (Lee et al., 2006). In line with previous findings (Arunachalam, Samuel, Marei, Ding, & Triggle, 2014), the present study showed more SA‐β‐gal‐positive cells in the HG‐treated group than in the control group; however, the HG‐treated cells significantly recovered with co‐treatment with CT. Furthermore, the number of SA‐β‐gal‐positive cells increased, and p‐Sirt1 expression and EC migration decreased, under HG conditions. However, the expression of p‐Sirt1 was increased by CT treatment. These results show that HG can promote EC senescence. Maeda, Hayashi, Mizuno, Hattori, and Kuzuya (2015) reported that persistent HG conditions increase SA‐β‐gal activity, further elucidating the possibility of replicative senescence.

In mammalian cells, the activation of Akt induces cell proliferation and survival, and the underlying mechanisms have been reported to regulate not only cell death directly but also several components of the cell cycle (Datta, Brunet, & Greenberg, 1999; Testa & Bellacosa, 2001). In agreement with previous findings (Servillo et al., 2013), we observed that the incubation of ECs with HG for 48 hr reduced cell proliferation by arresting the cell cycle, thereby increasing the percentage of cells in the G0/G1 and G2/M phases. Increased levels of cyclin‐dependent kinase inhibitors, such as p16 and p21, are frequently observed in senescent or aging cells (Stein, Drullinger, Soulard, & Dulić, 1999). Similarly, a considerable increase in p53, p21, and Rb levels was noted in ECs exposed to HG (30 mmol/L) for 48 hr. However, the change in p16 expression was not significant (data not shown). Mitogen‐activated protein kinases (MAPKs), a family of serine/threonine kinases that can be divided into three subgroups, ERK, c‐Jun N‐terminal kinase, and p38 MAPK, regulate cell proliferation and differentiation caused by various cell stresses. Moreover, the upregulation of p38 activates the p53/p21/Rb pathway (Iwasa, Han, & Ishikawa, 2003). The underlying mechanistic changes induced by treatment with high levels of glucose result in the increased expression of p53, which subsequently leads to the increased expression of downstream p21 levels (Cao et al., 2019; Wu, Lee, Bobadilla, Duan, & Liu, 2017). Consistent with the findings of previous studies, our study showed that CT delays the senescence of HG‐treated ECs, primarily via the regulation of the Akt/p53/p21 pathway.

## 5 CONCLUSIONS

Our study is the first to report that CT mediates HG‐induced EC senescence via its action on the Akt/p53/p21 pathway. CT may be a functional agent for the protection of ECs owing to its anti‐senescence effects.

## CONFLICT OF INTEREST

The authors declare that they do not have any conflict of interest.

## ETHICAL STATEMENT

This study does not involve any human or animal testing.
